# Supplementary material for: Feed Regime Slightly Modifies the Bacterial but Not the Fungal Communities in the Intestinal Mucosal Microbiota of Cobia Fish (Rachycentron canadum)
Source: Microorganisms. 2023 Sep 14;11(9):2315. doi: 10.3390/microorganisms11092315 (PMC10535204; doi:10.3390/microorganisms11092315)
Supplement: Supplementary file 1 [file microorganisms-11-02315-s001.zip › microorganisms-2544621-supplementary.pdf]

SUPPLEMENTARY TABLES:

Supplementary Table S1. Fish diet compositions.

Diet	FF (Formulated feed)	FFP (Frozen Fish Pieces)
Description	Commercial diet, Skretting-GISIS®	mixture of local fish: *Auxis* sp., *Scomber japonicus,* and *Opisthonema* sp.
Diameter	7 mm	-
Protein	40.0%	18.1%
Lipids	13.0%	5.8%
Moisture	11.0%	73.5%
Fiber	3.0%	0.0%
Reference	Skretting, 2023	Apolinario Castillo, 2017

Supplementary Table S2. Fish sampled for the microbiota study.

	Fish information					Information of intestinal samples		16S rARN (V3-V4) sequencing		ITS2 region sequencing	
**Sampling date**	**Feed**	**Tank**	**N°**	**Sex**	**Weight (g)**	**Sample code**	**DNA concentration (ng/µL)**	**QC and amplification in Novogene**	**Analyzed in this study**	**QC and amplification in Novogene**	**Analyzed in this study**
17/2/2021	FF	B1	1	IND	236.20	C7	172.9	pass	yes	pass	yes
			2	IND	447.20	C11	89.5	pass	yes	pass	No, <400 clean reads
			3	IND	418.60	C9	67.7	pass	yes	pass	yes
			4	IND	415.60	C13	23.1	pass	yes	pass	yes
		B2	5	IND	250.60	C10	70	pass	yes	pass	yes
			6	IND	374.80	C12	28.5	pass	yes	pass	yes
			7	IND	448.40	C8	210.4	pass	yes	pass	yes
	FFP	B3	8	IND	447.20	C15	80.3	pass	yes	pass	No, <400 clean reads
			9	IND	463.60	C16	152.9	pass	No, <400 clean reads	pass	yes
			10	IND	497.70	C17	145.7	pass	**yes**	fail	**no**
			11	IND	497.60	C18	90.9	pass	yes	pass	yes
			12	IND	502.80	C19	20.7	pass	**yes**	fail	**no**
19/2/2021		B4	13	F	5200.00	C33	102.1	pass	yes	pass	yes
			14	M	4780.00	C35	329.2	pass	yes	pass	yes
26/2/2021			15	M	5140.00	C39	167.4	pass	yes	pass	yes
			16	M	5360.00	C41	241.7	pass	yes	pass	yes
							**Total sample analyzed**		**15**		**12**

Feed: FF, formulated feed; and **FFP**, frozen fish pieces. **Sex: IND**, indeterminate; **F**, female; and **M,** male. **QC:** quality control.

Supplementary Table S3. Bacterial counts and relative abundance. Phyla included in this table had a prevalence and detection threshold greater than 90% and 0.05%, respectively. Genera included in this table had a prevalence and detection threshold greater than 95% and 0.05%, respectively.

		FF GROUP									FFP GROUP										TOTAL	
**Taxa**	**Sample**	**C10**	**C11**	**C12**	**C13**	**C7**	**C8**	**C9**	**Counts**	**Relative abundance %**	**C15**	**C17**	**C18**	**C19**	**C33**	**C35**	**C39**	**C41**	**Counts**	**Relative abundance %**	**Total counts**	**Relative abundance %**
	Proteobacteria	50364	50441	50541	49715	51583	51519	50957	355120	**96,28**	52277	51322	51281	51371	48705	50666	50638	50859	407119	**96,58**	762239	**96,44**
	Firmicutes	905	542	586	1371	443	510	523	4880	**1,32**	159	449	289	369	2982	443	207	303	5201	**1,23**	10081	**1,28**
	Actinobacteriota	679	817	689	590	150	113	442	3480	**0,94**	117	470	615	413	365	655	880	636	4151	**0,98**	7631	**0,97**
	Acidobacteriota	165	260	200	120	136	98	119	1098	**0,30**	0	83	104	138	176	97	306	110	1014	**0,24**	2112	**0,27**
	Gemmatimonadota	117	140	127	204	70	54	65	777	**0,21**	9	47	110	135	89	112	143	201	846	**0,20**	1623	**0,21**
Phylum	Myxococcota	119	148	132	242	55	57	101	854	**0,23**	0	96	81	49	60	99	190	171	746	**0,18**	1600	**0,20**
	Bacteroidota	136	119	165	196	56	65	88	825	**0,22**	82	27	40	35	21	11	45	58	319	**0,08**	1144	**0,14**
	Verrucomicrobiota	115	116	125	94	63	43	26	582	**0,16**	0	53	59	65	50	47	117	139	530	**0,13**	1112	**0,14**
	Nitrospirota	33	45	50	39	20	7	26	220	**0,06**	0	31	4	18	12	13	35	52	165	**0,04**	385	**0,05**
	Others	0	11	28	3	0	0	42	84	**0,02**	9	10	0	0	4	85	20	56	184	**0,04**	268	**0,03**
	Total	52690	52690	52690	52690	52690	52690	52690	368830	**100,00**	52690	52690	52690	52690	52690	52690	52690	52690	421520	**100,00**	790350	**100,00**
	Photobacterium	43850	37839	45535	47310	50000	46623	48778	319935	**86,74**	48938	43367	47114	48623	15551	45120	42487	6799	297999	**70,70**	617934	**78,18**
	Vibrio	1837	6911	3029	797	505	3024	688	16791	**4,55**	1387	4719	1037	1127	29193	2514	4463	41143	85583	**20,30**	102374	**12,95**
	Catenococcus	2746	4308	617	254	462	1180	820	10387	**2,82**	1314	1455	625	791	2929	1386	2226	1407	12133	**2,88**	22520	**2,85**
	Clostridium sensu stricto 1	36	3	38	665	33	170	23	968	**0,26**	34	340	180	253	2901	280	152	212	4352	**1,03**	5320	**0,67**
Genus	*Enterovibrio*	324	178	212	192	201	210	161	1478	**0,40**	144	316	1702	292	403	464	190	297	3808	**0,90**	5286	**0,67**
	Gemmatimonas	117	138	127	204	70	54	65	775	**0,21**	9	47	110	135	89	112	143	201	846	**0,20**	1621	**0,21**
	Faecalibacterium	129	69	145	164	125	99	119	850	**0,23**	15	22	24	30	10	17	25	20	163	**0,04**	1013	**0,13**
	Cutibacterium	16	14	22	45	4	5	10	116	**0,03**	66	36	54	39	52	70	60	51	428	**0,10**	544	**0,07**
	Others	51	82	58	47	0	9	54	301	**0,08**	1	14	45	0	2	66	65	36	229	**0,05**	530	**0,07**
	Total	52690	52690	52690	52690	52690	52690	52690	368830	**100,00**	52690	52690	52690	52690	52690	52690	52690	52690	421520	**100,00**	790350	**100,00**

FF group, fish fed formulated feed, and **FFP group,** fish fed frozen fish pieces.

Supplementary Table S4. Fungal counts and relative abundance. All phyla were included in this table. Genera included had a prevalence and detection threshold greater than 70% and 0.5%, respectively.

		**FF GROUP**								**FF GROUP**								**TOTAL**	
**Taxa**	**Sample**	**C10**	**C12**	**C13**	**C7**	**C8**	**C9**	**Counts**	**Relative abundance %**	**C16**	**C18**	**C33**	**C35**	**C39**	**C41**	**Counts**	**Relative abundance %**	**Total counts**	**Relative abundance %**
Phylum	p__Ascomycota	1897	1582	1743	1686	1675	1749	10332	**89,32**	1588	1863	1354	1667	1719	1836	10027	**86,68**	20359	**88,00**
	p__Basidiomycota	31	346	185	237	239	149	1187	**10,26**	328	58	574	241	178	79	1458	**12,60**	2645	**11,43**
	p__Mortierellomycota	0	0	0	3	0	30	33	**0,29**	0	0	0	16	10	4	30	**0,26**	63	**0,27**
	p__Aphelidiomycota	0	0	0	2	14	0	16	**0,14**	0	0	0	0	21	9	30	**0,26**	46	**0,20**
	p__Glomeromycota	0	0	0	0	0	0	0	**0,00**	12	0	0	0	0	0	12	**0,10**	12	**0,05**
	p__Olpidiomycota	0	0	0	0	0	0	0	**0,00**	0	0	0	4	0	0	4	**0,03**	4	**0,02**
	p__Rozellomycota	0	0	0	0	0	0	0	**0,00**	0	7	0	0	0	0	7	**0,06**	7	**0,03**
	total	1928	1928	1928	1928	1928	1928	11568	**100,00**	1928	1928	1928	1928	1928	1928	11568	**100,00**	23136	**100,00**
Genus	Unknown	200	552	998	1100	1031	975	4856	**41,98**	1457	1470	1750	811	686	665	6839	**59,12**	11695	**50,55**
	g__Debaryomyces	1420	627	16	77	11	226	2377	**20,55**	29	86	9	213	399	29	765	**6,61**	3142	**13,58**
	g__Ascobolus	28	15	244	183	268	105	843	**7,29**	19	18	1	0	106	59	203	**1,75**	1046	**4,52**
	g__Saccharomyces	148	401	0	47	33	119	748	**6,47**	67	86	12	101	3	96	365	**3,16**	1113	**4,81**
	g__Coprinopsis	2	19	31	67	124	23	266	**2,30**	0	6	0	5	26	39	76	**0,66**	342	**1,48**
	g__Cladosporium	2	40	54	29	17	0	142	**1,23**	41	105	29	44	88	12	319	**2,76**	461	**1,99**
	g__Yarrowia	33	51	0	42	8	0	134	**1,16**	6	32	14	64	0	38	154	**1,33**	288	**1,24**
	g__Apiotrichum	2	7	13	40	38	0	100	**0,86**	15	0	0	18	12	10	55	**0,48**	155	**0,67**
	g__Malassezia	0	1	22	11	16	0	50	**0,43**	4	7	25	40	46	4	126	**1,09**	176	**0,76**
	Other	93	215	550	332	382	480	2052	**17,74**	290	118	88	632	562	976	2666	**23,05**	4718	**20,39**
	total	1928	1928	1928	1928	1928	1928	11568	**100,00**	1928	1928	1928	1928	1928	1928	11568	**100,00**	23136	**100,00**

FF group, fish fed formulated feed, and **FFP group,** fish fed frozen fish pieces.

SUPPLEMENTARY FIGURES:

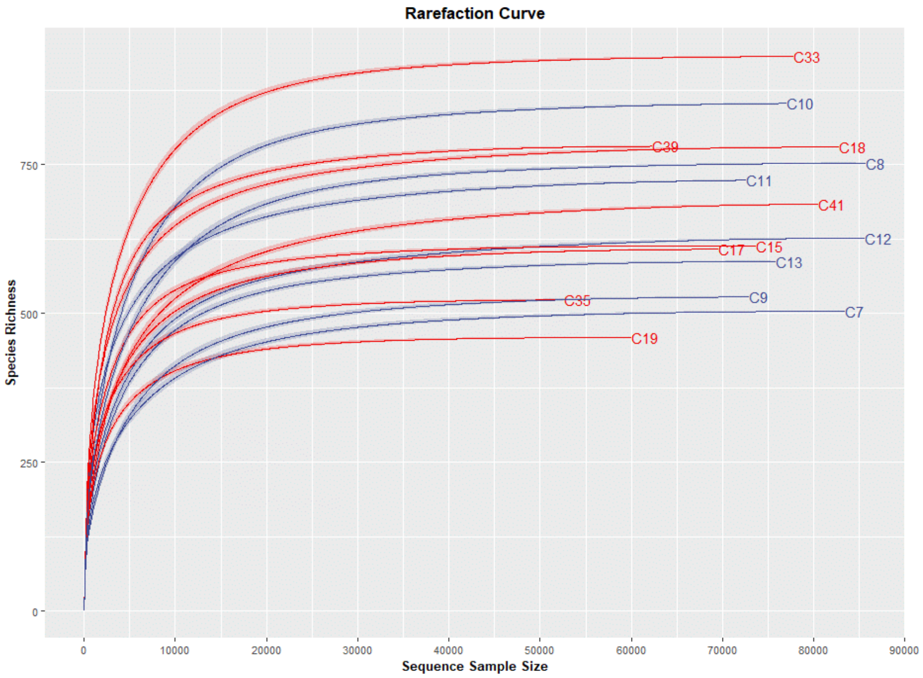

Supplementary Figure S1. Rarefaction curves showing the richness of the bacterial communities in the gut of cobia (*Rachycentron canadum*) fed with FF, formulated feed (blue line), and FFP, frozen fish pieces (red line).

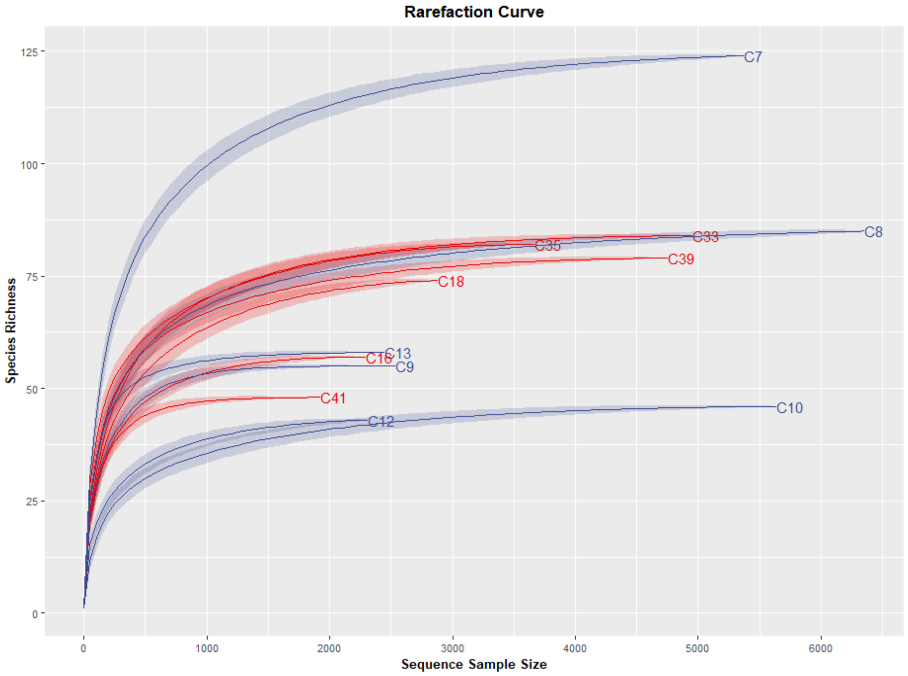

Supplementary Figure S2. Rarefaction curve showing the richness of the mycobiota in the gut of cobia (*Rachycentron canadum*) fed with FF, formulated feed (blue line), and FFP, frozen fish pieces (red line).

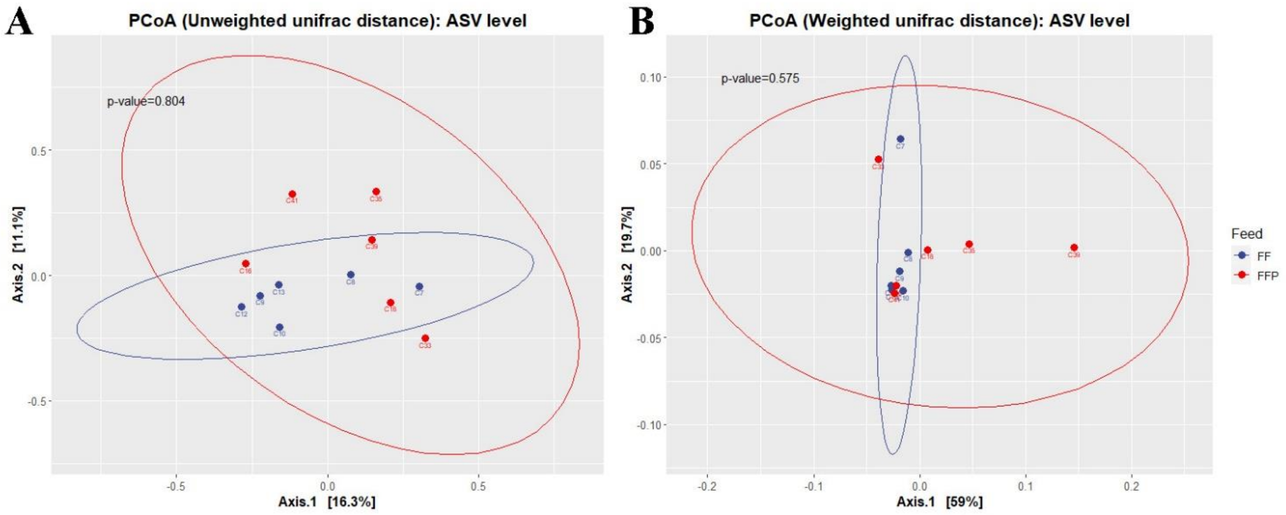

Supplementary Figure S3. PCoA analysis of cobia (*Rachycentron canadum*) intestinal mycobiota fed with formulated feed (FF) and frozen fish pieces (FFP) using ASVs. **A)** Unweighted Unifrac distance, non-significant distance between groups (p = 0.804), where the principal components explain 14.5 and 12.7% of data variance; and **B)** Weighted Unifrac distance, non-significant distance between groups (p = 0.575), where the principal components explain 42.1 and 20.3% of data variance. The ellipses represent 95% confidence level groups for a multivariate normal distribution.

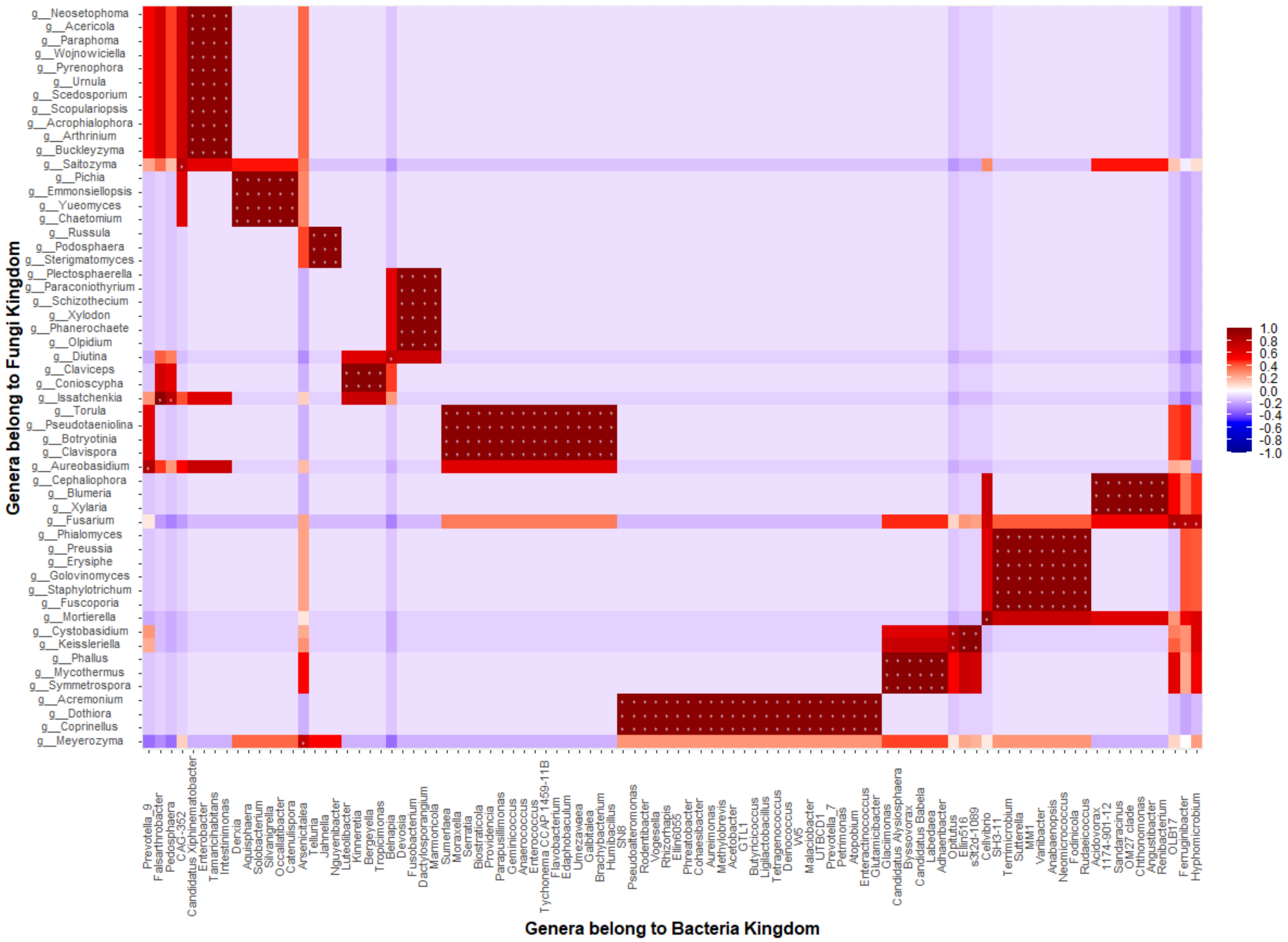

Supplementary Figure S4. Heatmap of Spearman's correlation (p≤0.05) between the abundance of genera belonging to fungal and bacterial communities of cobia (*Rachycentron canadum*). Crosses (+) indicate significant correlations.

A) Bacterial communities

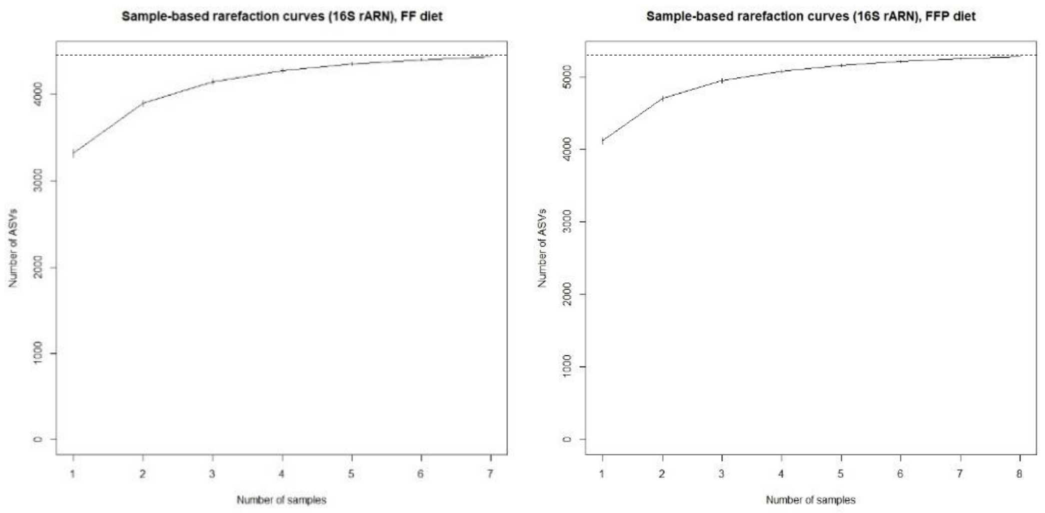

B) Fungal communities

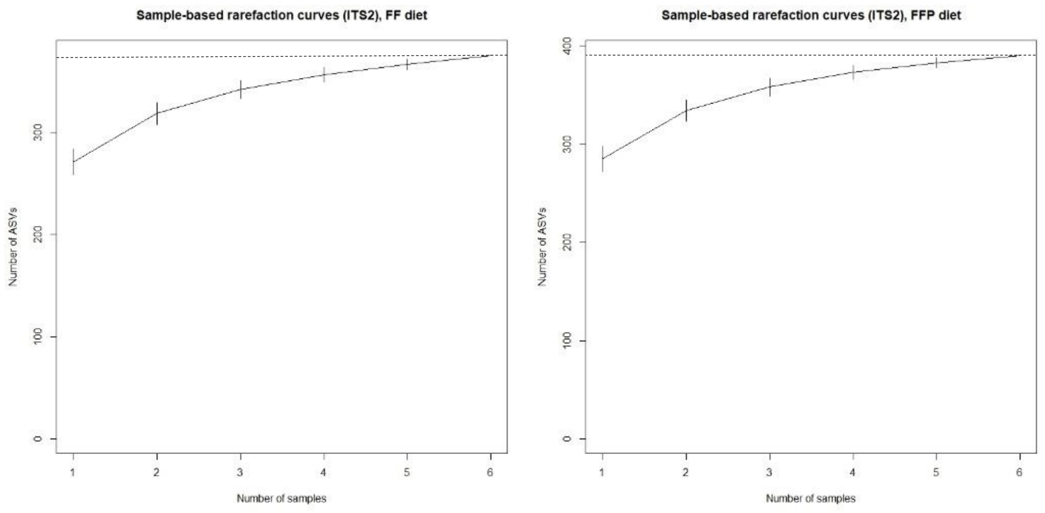

Supplementary Figure S5. ASV accumulation curves with sample size per treatment, showing the extent to which, each additional sample increases total number of ASVs detected per treatment for **A)** bacterial and **B)** fungal communities of cobia (*Rachycentron canadum).* Horizontal dotted lines indicate the asymptote of the curve.
